# *Toxoplasma gondii* Myocarditis after Adult Heart Transplantation: Successful Prophylaxis with Pyrimethamine

**DOI:** 10.1155/2012/853562

**Published:** 2012-11-01

**Authors:** Tania Mara V. Strabelli, Rinaldo Focaccia Siciliano, Silvia Vidal Campos, Jussara Bianchi Castelli, Fernando Bacal, Edimar A. Bocchi, David E. Uip

**Affiliations:** ^1^Infection Control Unit, Heart Institute (Incor-HCFMUSP), University of São Paulo Medical School, São Paulo, SP, Brazil; ^2^Lung Transplant Unit, Heart Institute (Incor-HCFMUSP), University of São Paulo Medical School, São Paulo, SP, Brazil; ^3^Pathology Unit, Heart Institute (Incor-HCFMUSP), University of São Paulo Medical School, São Paulo, SP, Brazil; ^4^Heart Transplant Unit, Heart Institute (Incor-HCFMUSP), University of São Paulo Medical School, São Paulo, SP, Brazil

## Abstract

*Toxoplasma gondii* primary infection/reactivation after solid organ transplantation is a serious complication, due to the high mortality rate following disseminated disease. We performed a retrospective study of all cases of *T. gondii* infections in 436 adult patients who had received an orthotopic cardiac transplant at our Institution from May 1968 to January 2011. Six patients (1.3%) developed *T. gondii* infection/reactivation in the post-operative period. All infections/reactivations occurred before 1996, when no standardized toxoplasmosis prophylactic regimen or co-trimoxazole prophylaxis was used. Starting with the 112th heart transplant, oral pyrimethamine 75 mg/day was used for seronegative transplant recipients whose donors were seropositive or unknown. Two patients (33.3%) presented with disseminated toxoplasmosis infection, and all patients (100%) had myocarditis. Five patients (83.3%) were seronegative before transplant and one patient did not have pre-transplant serology available. Median time for infection onset was 131 days following transplantation. Three patients (50%) died due to toxoplasmosis infection. After 1996, we did not observe any additional cases of *T. gondii* infection/reactivation. In conclusion, toxoplasmosis in heart allographs was more frequent among seronegative heart recipients, and oral pyrimethamine was highly effective for the prevention of *T. gondii* infection in this population.

## 1. Background

Toxoplasmosis is a zoonotic disease of worldwide distribution, with a high incidence in certain countries. Humans are usually infected through ingestion of oocysts that are present in contaminated water or soil, or by eating undercooked meat with tissue cysts. Although the typical course of acute infection is a benign febrile syndrome in healthy patients, severe disease occurs in hosts with immune system impairment, including transplant and AIDS patients. Transplant recipients who are seronegative for *Toxoplasma gondii* are at particular risk of developing a severe toxoplasmosis infection upon receipt of an organ from a seropositive donor [1].

In this study, we studied six cases of *T. gondii* myocarditis following heart transplantation before routine use of pyrimethamine prophylaxis in our institution.

## 2. Patients and Methods

We performed a retrospective study of all cases of *T. gondii* infections in adult patients who had received an orthotopic cardiac transplant at our institution from May 1968 to January 2011. The Heart Institute (Incor-HCFMUSP) is a tertiary cardiology care hospital in São Paulo, Brazil, with a heart transplant program that was founded in 1964. Until 1996, there was no standardized toxoplasmosis prophylactic regimen. Starting with the 112th heart transplant, oral pyrimethamine 75 mg/day was used for seronegative transplant recipients whose donors were seropositive or unknown. This regimen was given from the first day after transplant and was maintained until the 100th day after transplant. *Pneumocystis jiroveci *pneumonia prophylaxis with cotrimoxazole was not used following heart transplant. We diagnosed toxoplasmosis by analyzing the histology of endomyocardial biopsies and/or necropsies, in which *Toxoplasma* infection was determined by morphology using hematoxylin and eosin, and immunohistochemistry using commercially available polyclonal rabbit antibody against *T. gondii* (produced by Chemicon International, Inc., Temecula, CA, USA). Patient medical charts from all histologically confirmed *T. gondii* infections were reviewed.

## 3. Results

**Figure 1:**
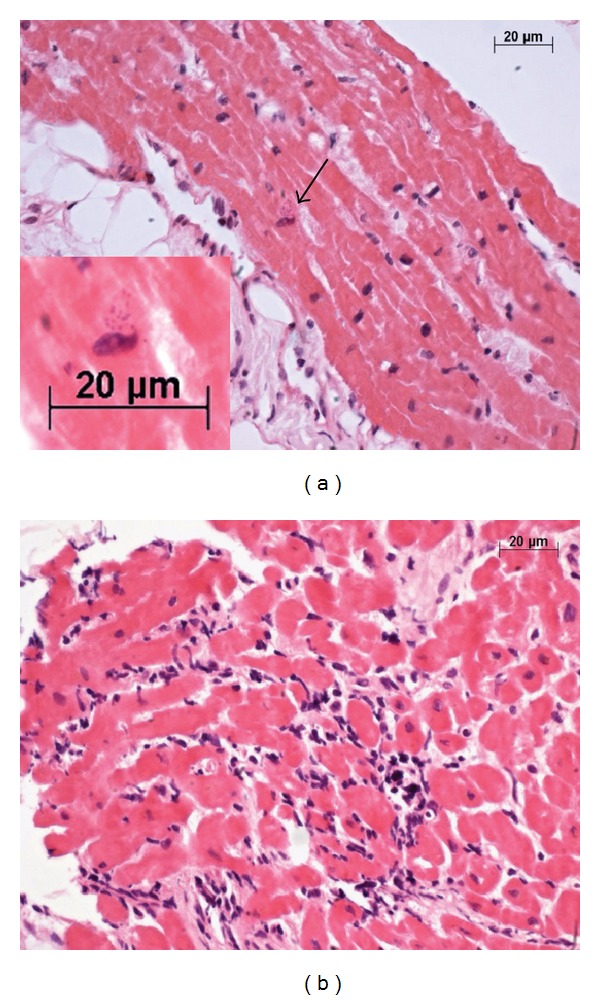
Photomicrographs of the endomyocardial biopsy. (a) In this region of the myocardium, a small *Toxoplasma* pseudocyst was noted in one cardiomyocyte (arrow). The inset shows amplification of this area, as indicated by the arrow. The presence of round corpuscles consistent with *T. gondii* tachyzoites near the cardiomyocyte nucleus was noted. (b) In another region of the myocardium within the same endomyocardial biopsy sample, there were moderate amounts of mononuclear infiltrates with aggression and destruction of the cardiomyocyte. This led to the misdiagnosis of rejection, even though it was subsequently determined that this case of myocarditis was caused by *T. gondii*. ((a) and (b) Hematoxylin and eosin stain with objective ×40 in all images and digital zoom at the inset).

**Figure 2:**
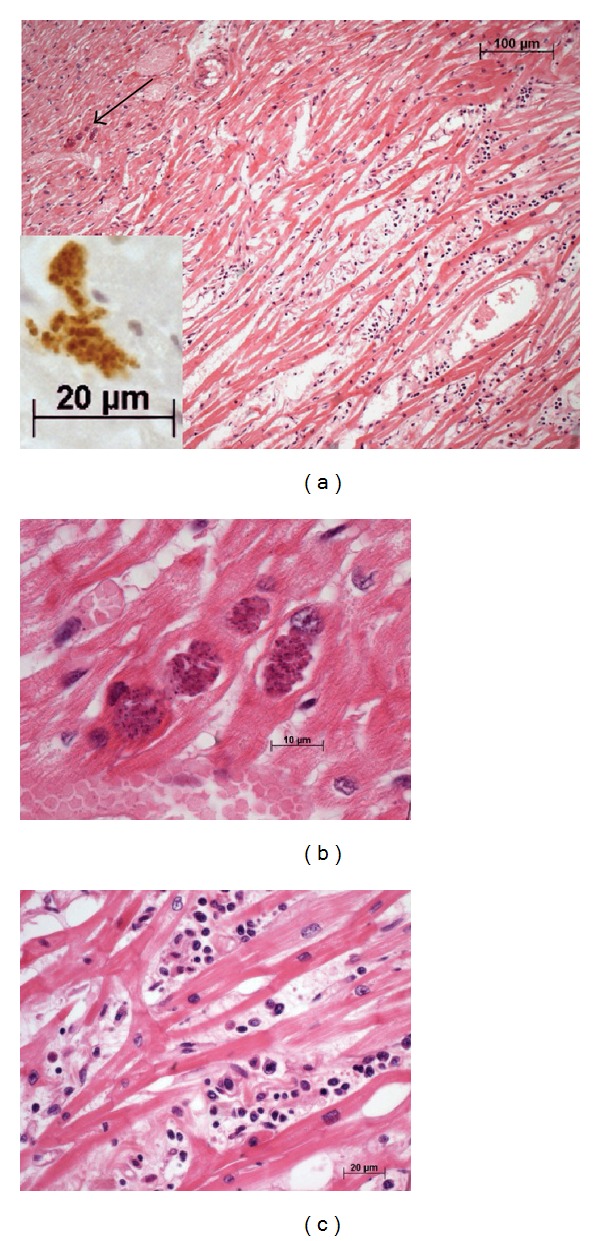
Photomicrographs of the myocardium in the necropsy. (a) In panoramic view, moderate to intense myocardial inflammation with edema and mononuclear cells is visible. Large *Toxoplasma* pseudocysts, indicated by the arrow, existed in a region without inflammatory infiltrate. This region is amplified in image (b). The inset shows amplification of the pseudocyst, with antibody specific for* T. gondii* highlighting the round corpuscles, which is consistent with the tachyzoites form of *T. gondii*. (b) The tachyzoites are still closed and hidden intracellularly. (c) A high magnification of the myocardium in an area of myocarditis. The infiltrate was composed of lymphocytes, plasma cells and histiocytes ((a), (b) and (c) Hematoxylin and eosin stain with objective ×10, ×100 oil and ×40, respectively; objective ×40 with digital zoom at the inset).

During the study period, 436 adult heart transplants were performed, and six patients (1.3%) developed *T. gondii* infection/reactivation in the postoperative period. All infections/reactivations occurred before 1996, and none of the patients received pyrimethamine prophylaxis. Demographic and clinical information are described in Table 1, and examples of histological examination are in Figures 1 and 2. All patients were seronegative for Chagas' disease. Two patients (33.3%) presented with disseminated toxoplasmosis infection, and all patients (100%) had myocarditis. Five patients (83.3%) were *Toxoplasma* seronegative before transplant and one patient did not have before transplant serology available. Median time for *Toxoplasma* infection onset was 131 days (mean = 69 days, range 21–534) following transplantation. No seroconversion was detected. Three patients (50%) died due to toxoplasmosis infection. After 1996, we did not observe any additional cases of *T. gondii* infection/reactivation.

## 4. Discussion

*T. gondii* primary infection/reactivation after solid organ transplantation is a serious complication, due to the high mortality rate after disseminated disease. Following transplant, the parasitic infection is clearly related to the type and duration of suppressive therapy [1]. In a multicenter, matched-case control study [2], the frequency of toxoplasmosis was significantly higher in heart recipients compared to the kidney and liver recipients. A negative serostatus prior to transplantation was the only independent risk factor for toxoplasmosis (odds ratio = 15.12 [95% confidence interval, 2.37–96.31]; *P* = .004). The central nervous system is mostly affected by this infection in AIDS patients [3]. Patients receiving heart transplants have a higher risk of developing *T. gondii-*induced  myocarditis when compared with other solid organ transplants. Campbell et al. reviewed tissue-invasive toxoplasmosis in noncardiac solid organ transplant recipients (kidney, liver, small bowel, and pancreas) and found that in 52 cases, 44 (85%) patients had disseminated disease, while only 19% developed myocarditis [4]. Patients with toxoplasmosis after heart transplantation have myocarditis that may cause allograph dysfunction in 75%; disseminated disease occurs in nearly 45% of them [5]. Mortality due to *Toxoplasma* infection is also high. In a kidney transplant patient cohort with 31 cases of Toxoplasmosis, 20 patients (64.5%) died [6]. In our study, we observed 50% mortality among the six infected patients. Death in most reported cases has been related to a delay in diagnosis and targeted treatment [2].

Endomyocardial biopsy (EMB) is a reliable way to identify and track rejection following heart transplantation, and in some cases, EMB may show the presence of infectious organisms, including *Toxoplasma* pseudocysts. Moreover, *Toxoplasma* is one of the most commonly opportunistic organisms diagnosed on EMB [7]. *Toxoplasma*-related myocarditis has a fugacious exudative or neutrophilic component occurring when the myocyte ruptures following *Toxoplasma* proliferation. This fugacious component becomes mononuclear shortly after rupture, which results in an increasingly difficult differential diagnosis of rejection. Therefore, in some cases, the lymphoid infiltrate may not be caused by transplant rejection. In addition, the pseudocysts are often present in non-inflamed myocardial tissue, that is, when there is no myocyte rupture and the microorganism remains hidden from the immune system and may go unnoticed, even by a very experienced pathologist. This occurred in one case in our series of patients, where the review of a biopsy showed a small *Toxoplasma* pseudocyst present in a non-inflamed area of the myocardial sample. The use of *T. gondii* immunohistochemistry could help with the differential diagnosis of seronegative patients with suspected acute rejections (Figures 1 and 2). Consequently, we would like to caution the pathologist to be careful in their interpretation of tissue samples, considering the difficulty in visualizing *Toxoplasma* pseudocysts, as well as the difficulty in differentiating the diagnosis of rejection when inflammation is induced. In our series, five of six *Toxoplasma* infection presented as cardiac failure without fever. In the first months following cardiac transplant, this clinical situation could be misdiagnosed as acute rejection, resulting in increased immunosuppression and inadvertent favor of *T. gondii* infection.

Although the combination of pyrimethamine and sulfadiazine is the most effective regimen in the prevention of *T. gondii* infection, monotherapy with pyrimethamine has been suggested after heart transplantation for prophylaxis [8, 9]. Other transplant programs that have been successful in preventing *Toxoplasma* infection include the use of trimethoprim/sulfamethoxazole (cotrimoxazole) during the post transplant period [10–12]. Cotrimoxazole is systematically used following kidney or liver transplants to prevent opportunistic infections, such as pneumocystosis, but there is no consistent evidence for its efficacy following heart transplantation. In a review of two transplant programs in the USA, Baran et al. found no evidence of toxoplasmosis postheart transplantation and proposed that *P. jiroveci* prophylaxis with cotrimoxazole is sufficient to prevent toxoplasmosis infection/reactivation [13]. Our heart transplant program does not recommend routine prophylaxis with cotrimoxazole for pneumocystosis prevention. In areas with a high prevalence of toxoplasmosis (50%–80% in adults), such as in Brazil [14], there is a low number of seronegative patients receiving heart transplants, and the prophylaxis could be individualized. Considering the adverse events related to universal *P. jiroveci* prophylaxis with cotrimoxazole (i.e., skin rashes or nephrotoxicity) and the low risk of pneumocystosis, we adopted the use of pyrimethamine as *Toxoplasma* prophylaxis in seronegative recipients. The reactivation of *T. gondii* within seropositive recipients is rare following heart transplantation [15]. We did not observe any incidents of *T. gondii* reactivation (IgG-positive recipients) in 43 years of cardiac transplant experience.

In conclusion, toxoplasmosis in heart allographs was more frequent among seronegative heart recipients, and oral pyrimethamine was highly effective for the prevention of *T. gondii* infection in this population.

## Figures and Tables

**Table 1:** Clinical and laboratorial data for six cases of *Toxoplasma* infection after cardiac transplantation without toxoplasmosis prophylaxis occurring before 1996.

Patient sex/age (years)	Toxoplasmosis serostatus D/R—IgG	Time from transplant to toxoplasmosis diagnosis (days)	Source of diagnosis	Signs/symptoms	Treatment (days)	In-hospital lethal outcome*
M/52	NA/Neg	21	EMB	Heart failure	S + P	Yes
F/61	NA/Neg	534	EMB	None	S + P	No
M/44	NA/Neg	70	EMB	Heart failure	S + P	No
M/66	NA/NA	69	Necropsy (CNS and heart)**	Heart failure and low-level consciousness	—	Yes
M/49	NA/Neg	25	EMB	Heart failure	S + P	No
F/49	NA/Neg	69	Necropsy (CNS, heart and lungs)	Heart and respiratory failure and low-level consciousness	—	Yes

Note: M: male; F: female; D/R: donor/recipient; NA: not available; Neg: negative; EMB: endomyocardial biopsy; CNS: central nervous system; S + P: sulfadiazine plus pyrimethamine.

*Survival after discharge not analyzed.

**The EMB was misdiagnosed as a rejection, and the patient subsequently received methylprednisolone 1 g/d for 3 days. At necropsy, the EMB was reviewed and the diagnosis of toxoplasmosis was determined.
